# Urban Area Detection in Very High Resolution Remote Sensing Images Using Deep Convolutional Neural Networks

**DOI:** 10.3390/s18030904

**Published:** 2018-03-18

**Authors:** Tian Tian, Chang Li, Jinkang Xu, Jiayi Ma

**Affiliations:** 1 Hubei Key Laboratory of Intelligent Geo-Information Processing, College of Computer Science, China University of Geosciences, Wuhan 430074, China; tiantian@cug.edu.cn; 2 Department of Biomedical Engineering, Hefei University of Technology, Hefei 230009, China; lichang@hust.edu.cn; 3 School of Automation, Huazhong University of Science and Technology, Wuhan 430074, China; hustxujinkang@gmail.com; 4 Electronic Information School, Wuhan University, Wuhan 430072, China

**Keywords:** urban area detection, remote sensing, very high resolution, deep convolutional neural networks

## Abstract

Detecting urban areas from very high resolution (VHR) remote sensing images plays an important role in the field of Earth observation. The recently-developed deep convolutional neural networks (DCNNs), which can extract rich features from training data automatically, have achieved outstanding performance on many image classification databases. Motivated by this fact, we propose a new urban area detection method based on DCNNs in this paper. The proposed method mainly includes three steps: (i) a visual dictionary is obtained based on the deep features extracted by pre-trained DCNNs; (ii) urban words are learned from labeled images; (iii) the urban regions are detected in a new image based on the nearest dictionary word criterion. The qualitative and quantitative experiments on different datasets demonstrate that the proposed method can obtain a remarkable overall accuracy (OA) and kappa coefficient. Moreover, it can also strike a good balance between the true positive rate (TPR) and false positive rate (FPR).

## 1. Introduction

Over the last few decades, urban area detection from very high resolution (VHR) remote sensing images has become more and more important for many applications, such as map updating, urban development analysis, military reconnaissance and disaster management [1,2,3,4,5,6]. Traditional methods were manually operated. However, it was time consuming to process images covering large regions due to their high resolution. Therefore, it is beneficial to develop automatic and real-time detection techniques for urban area detection in large-scale datasets of VHR remote sensing images [7,8,9,10].

Recently, various techniques have been proposed to detect the urban areas automatically, which can be roughly divided into two categories, i.e., supervised and unsupervised. Most of the unsupervised urban area detection methods are based on the texture and structure characteristics of the VHR remote sensing images [2], which generally involve feature extraction and matching steps [11]. Sirmacek et al. used the scale-invariant feature transform (SIFT) to obtain keypoints and then extracted urban areas based on multiple subgraph matching [12]. However, the SIFT feature is computationally expensive, which limits the efficiency of their approach. To address this issue, they subsequently proposed a new method based on local feature points using Gabor filters together with spatial voting [13], which can deal with dynamic urban areas. Kovacs et al. further improved the detection performance by using the Harris-based feature point set and adaptive orientation-sensitive voting technique [14]. Alternatively, Tao et al. located the candidate regions by incorporating the extracted corners and modeled the candidate regions with a texture histogram, then the non-urban regions were removed by spectrum clustering and graph cuts [15]. However, it needs to cooperate with several other images to accomplish the detection. Methods incorporating Harris corner voting and other techniques also can be found in literature [2,16], in which guided filtering and Cauchy graph embedding optimization are used, respectively. In fact, their skeleton pipelines still follow the traditional Harris-based feature and spatial voting model. More approaches concerning unsupervised urban area detection appear in environmental remote sensing research [17,18,19], in which various indices such as NDVI (Normalized Difference Vegetation Index) and NDBI (Normalized Difference Built-up Index) are taken to assess the urban land coverage. However, these research works are usually done on multispectral moderate or low resolution remote sensing images, which are not suitable for VHR remote sensing images. In brief, the above-mentioned unsupervised urban area detection methods do not have the training process; therefore, the detection results are typically not accurate enough.

The other group of methods are supervised detection methods, which require a priori training data to conduct the detection. Benediktsson et al. proposed a framework for urban area detection from VHR remote sensing images based on feature extraction (or feature selection) and classification [20]. First, differential morphological profiles are built, which can describe the structural information of images. Second, feature extraction or feature selection is applied to the differential morphological profiles. Finally, a neural network is adopted to classify the features obtained from the second step. Bruzzone et al. proposed to classify the urban areas by means of support vector machine (SVM) [21]. Fauvel et al. proposed an adaptive decision fusion for the classification of urban remote sensing images by combining multiple classifiers based on fuzzy sets and possibility theory [22]. Weizman et al. employed the concept of visual words (feature-free image representation) to extract the urban areas [23]. Visual words possess the advantages of being not limited by a predefined set of features and being robust to changes in scene and illumination effects, which have been widely used in the areas of image analysis and computer vision, such as image classification, clustering and retrieval. Li et al. used multi-kernel learning to incorporate multiple features [24]. Hu et al. used multi-scale features to build a supervised framework for built-up area detection [25]. Alternatively, Zhang et al. employed different indices and object-oriented classification to distinguish urban area from non-urban areas. Although these supervised methods have achieved acceptable performance in urban area detection, they are dependent on manually-designed features. These feature extraction processes are not able to extract rich features from various data automatically, which involves different levels of abstract patterns ranging from locally low-level information (e.g., textures and edges) to more semantic descriptions (e.g., object parts of mid-level descriptors and whole objects for the high level) [26].

In the recent past, deep convolutional neural networks (DCNNs) have become very popular in the applications of pattern recognition, computer vision and remote sensing [27,28,29]. DCNNs usually contain a number of convolutional layers and a classification layer, which can learn deep features from the training data and exploit spatial dependence among them. Krizhevsky et al. trained a large DCNN to classify 1.2 million high-resolution images in ImageNet and obtained superior image classification accuracy [30]. Ding et al. combined DCNNs with three types of data augmentation operations to improve the performance of synthetic aperture radar target recognition and obtained the best performance among the competitors [31]. Chen et al. proposed hybrid DCNNs for vehicle detection in high-resolution satellite images [32]. Penatti et al. evaluated the generalization power of DCNNs in two new scenarios: aerial and remote sensing image classification [33], and achieved very high accuracy of approximately 99.43% on the UC Merced dataset of aerial images. Nogueira et al. presented an analysis of three strategies for exploiting the power of DCNNs in different scenarios for remote sensing scene classification: full training, fine tuning and using DCNNs as feature extractors [26]; and the results demonstrated that fine tuning tends to be the best performing strategy. Sherrah et al. applied DCNNs to semantic labeling of high-resolution remote sensing data [34], which yielded state-of-the-art accuracy for the ISPRS Vaihingen and Potsdam benchmark datasets. The above-mentioned successful works have demonstrated the powerful abilities of DCNNs in a wide range of computer vision and pattern recognition applications.

In similar studies, deep neural networks were used in built-up area detection of SAR images [35] and detection of informal settlements [36]. To the best of our knowledge, little work based on DCNNs has been carried out on urban area detection tasks of VHR images. In this paper, we propose a new urban area detection method based on the DCNNs, which can extract rich features from training data automatically and efficiently. In our pipeline, pre-trained DCNNs are employed to extract deep features on the training data first, and then, *K*-means clustering is applied on the feature vectors to produce the visual words, which will construct the visual dictionary for the following detection steps. Subsequently, each patch of the training image is assigned to the nearest visual word, and we can obtain two word frequency histograms for the urban areas and the non-urban areas, respectively. Finally, for an arbitrary new patch, the probability that it belongs to an urban area is calculated with the Bayes’ rule, and we can determine whether it belongs to the urban area based on the pre-set threshold.

The main contribution of this work lies in the introduction of DCNNs into urban area detection of VHR remote sensing images. To the best of our knowledge, the urban area detection using visual words based on DCNNs has not yet been studied. The qualitative and quantitative experiments on real VHR remote sensing images demonstrate that, compared to other state-of-the-art approaches, the proposed method achieves the best overall accuracy (OA) and kappa coefficient. In addition, it can also strike a good balance between the true positive rate (TPR) and the false positive rate (FPR).

## 2. Proposed Method

**Figure 1:**
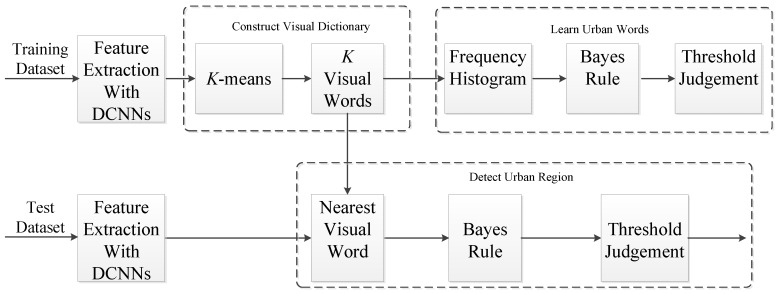
Algorithm flow of our proposed urban area detection method.

In this section, we first give a brief introduction of the DCNNs and then describe the proposed urban area detection method based on the DCNNs, which is mainly composed of three parts: constructing a visual dictionary based on DCNNs, learning urban words from labeled images and detecting urban regions in a test image. The algorithm flow of our proposed urban area detection method is shown in Figure 1.

For the task of urban area detection in VHR remote sensing images, it is not appropriate to extract features for every single pixel. Since the pixels usually differ from each other greatly even if they belong to the same area, pixel-based features with poor consistency will decrease the detection performance. Moreover, if each pixel is taken as a sample instead of a patch, then the large number of samples and heavy computational load are beyond our affordability. For the above-mentioned reasons, we adopt a simple and efficient preprocessing method to deal with both the training and testing VHR remote sensing images. In detail, we segment the images into different n×n non-overlapping patches and label the different image patches to urban area or non-urban area for the training images. For the test images, we also do the segmentation in the same way and detect the urban area with our proposed method.

### 2.1. Deep Convolutional Neural Networks

**Figure 2:**
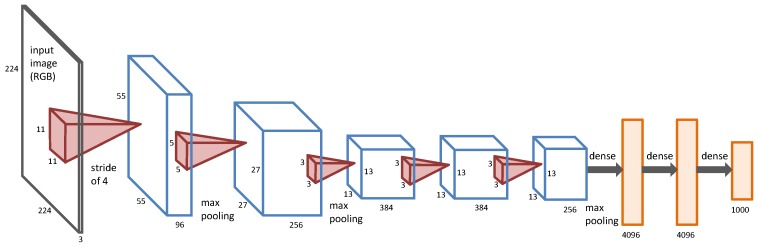
An illustration of the architecture of the DCNNs proposed by Alex et al. [30].

DCNNs are hierarchical models, which were first proposed by LeCun et al. [37] to overcome the problems of fully-connected deep neural networks, and they have obtained state-of-the-art performances for many computer vision and pattern recognition applications. Recently, by using fast GPUs, DCNNs were further used to solve the large-scale image classification problem [30,38]. In this paper, we adopt the architecture of DCNNs proposed by Alex et al. [30], which is shown in Figure 2.

To learn features with shift and distortion invariance, convolutional layers topologically encode spatial correlation by local receptive fields, and each feature map replicates the same features on the local receptive fields all over the input by feature pooling and shared weights. Shift and distortion invariance means to enforce the training samples to share similar features before and after shift and distortion. The spatial coverage of a filter with respect to the source image is called its receptive field, for example the receptive fields of the first and the second layers of the DCNNs in Figure 2 are 11×11 and 5×5, respectively. DCNNs usually have three kinds of layers: convolutional layers, pooling layers and fully-connected layers [39]. Convolutional layers work like a filter bank, which can produce convolutional information of the input image. The first convolutional layer works on a small window, which can only learn local low-level features. It is able to learn more robust and deep features from low-level features when the convolutional layer goes deeper. Pooling layers reduce the spatial size of the input layer and enable translation invariance, where the max-pooling operator is widely used for many applications. Fully-connected layers usually appear in the last few layers of the DCNNs, which connect all neurons in the previous layer, and features can be further summarized to produce deep features for subsequent applications.

For the urban area detection task of VHR remote sensing images, we use the feature extraction part of the pre-trained DCNNs to describe n×n non-overlapping patches. To be specific, the softmax layers are removed, and convolutional and pooling layers are preserved. The underlying reason is that labeled data are often very difficult and expensive to obtain, and pre-training can prevent overfitting and obtain better generalization when the number of labeled samples is small. Deep features can be transferred from natural images to remote sensing images, which has been verified in many works [26]. With the support of the abundant training data of ImageNet (1.2 million images and 1000 distinct classes), pretrained DCNNs have acquired a sufficient ability of image deep feature extraction and have shown remarkable results in many applications, such as human attribute detection [40], scene retrieval [41], robotics [42] and remote sensing [43,44,45,46,47]. Furthermore, it is very simple to adopt the pre-trained DCNNs as feature extractors since there is no training or tuning needed. This means the computing time of the feature extraction process can be reduced significantly, or this process can work well on small samples, as well. Therefore, we adopt the pre-trained DCNNs to extract the features of different n×n non-overlapping patches, and the extracted deep features can be used to construct the visual dictionary for urban area detection of VHR remote sensing images.

### 2.2. Constructing a Visual Dictionary Based on DCNNs

Since we have extracted a great number of deep features from different patch samples, we must analyze the latent patterns of these features that belong to urban or non-urban patches. The amount of different features becomes the first barrier for us to explore the inherent characteristics. In order to condense the feature set and capture the principle features, we employ the visual words model to process the deep features and construct a visual dictionary.

The visual words model, which derives from image classification, has been successfully applied in many image processing fields. Actually, the visual words are image patches that carry the most distinct characteristics. They are the clustering centroids of the whole set of all image patches, which are able to approximately represent any patch of the source image. The entirety of the visual words constitutes a visual dictionary, which carries only the refined and dissimilar features. By using the statistics of the occurrence of each word, we can easily represent an image and an object (within an image), namely classification tasks have to be implemented as a matter of fact.

**Figure 3:**
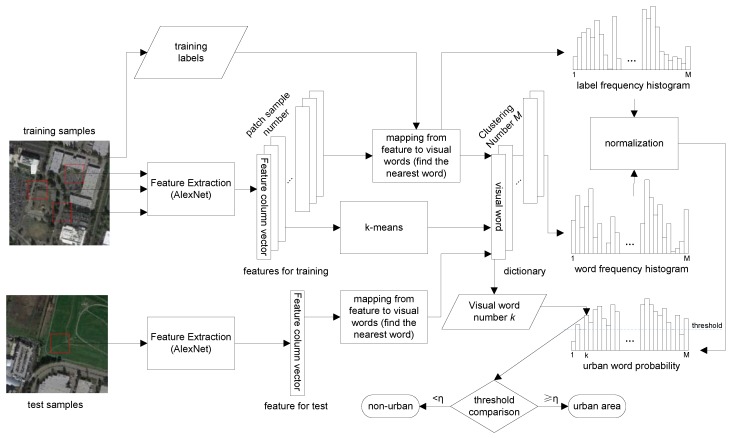
Details of each step in our proposed method.

In this paper, we first use the DCNNs to extract deep features from VHR remote sensing images and then randomly select a portion of patches to do the clustering (a convention in similar methods, mainly by the consideration of computation limits) with *K*-means [48]. Specifically, each training image is divided into n×n non-overlapping patches, and each patch is normalized by subtracting the patch mean. For each patch, we use the Caffe framework [38] to obtain the feature vector, where the parameters are set the same as those of the trained ones from the ImageNet database using the Caffe framework. The *K*-means clustering method is subsequently employed to cluster the features into *M* groups, and the clustering centroids are denoted by the visual words, which construct the final visual dictionary (Figure 3).

### 2.3. Learning Urban Words

Based on the above visual dictionary, urban and non-urban areas can be modeled with the frequency occurrence histograms of the dictionary words. Figure 3 illustrates the detailed methods of visual dictionary construction, urban words learning and urban regions detection. Since urban and non-urban areas are labeled on the training images, we first normalize and obtain the feature vector for each patch by the DCNNs described above and then assign each patch to the nearest dictionary word. By every mapping from feature to visual word of each training patch, we can establish two frequency histograms to record the distributions of labels (we choose one for urban and zero for non-urban, for the sake of simple histogram construction and convenient urban area detection) and dictionary words, respectively. After normalization, the two frequency histograms can be combined and regarded as the discrete distribution $P_{\mathrm{urban}}\left(.\right)$ of the visual words in the urban area (the complementary histogram will represent the distribution of non-urban area; however, since the task is a binary classification problem, the presence of the non-urban histogram is unnecessary here). Therefore, given an arbitrary patch, the probability that it belongs to an urban area can be obtained by adopting Bayes’ rule:

(1) $$P\left(\mathrm{urban}|u\right)=\frac{\alpha P_{\mathrm{urban}}\left(u\right)}{\alpha P_{\mathrm{urban}}\left(u\right)+\left(1-\alpha \right)P_{\mathrm{non}-\mathrm{urban}}\left(u\right)},$$

where *u* is an urban word and α is the prior probability of a patch that belongs to an urban area. Thus, we get a group of “urban words” that can best discriminate urban areas from non-urban areas when satisfying P(urban|u)≥η with η being a threshold.

### 2.4. Detecting Urban Regions in a Test Image

Detecting urban areas is actually a segmentation or a classification problem. Given a test image, we first get patches from the grid, repeat the same normalization and feature extraction process and then assign each patch to the nearest dictionary word. The probability that an arbitrary patch belongs to an urban area can be obtained by Equation (1). If P(urban|u)≥η, then the patch is labeled as the urban area. Since the spatial information is very important in remote sensing image representation, it is necessary to take such information into consideration in our method. To get a global smooth decision on urban areas, we adopt the post-processing strategy in [23] to incorporate spatial consistency, which is based on the morphological operator and is quite time efficient compared to traditional methods using the Markov random field model. We select a 5×5 window around each patch and obtain the proportion of urban area among these 25 patches. If more than half of these patches belong to the urban area, then this patch is determined as an urban area.

## 3. Experiments

In order to evaluate the performance of our algorithm, we conduct experiments on two datasets and use a number of state-of-the-art urban area detection methods for comparison, including visual word region detection (VWRD) [23], PCA + SVM, DCNNs + SVM, local feature points and spatial voting [13], texture histogram and spectral clustering [15], Cauchy graph embedding optimization [16] and convolutional neural network (CNN) [36]. The VWRD, PCA + SVM, DCNNs + SVM and CNN are supervised detection methods, while the others are unsupervised detection methods. In the following, we first provide the datasets and evaluation criteria used, followed by the discussion of the parameter settings. Subsequently, we present both the qualitative and quantitative results of different methods on the datasets.

### 3.1. Datasets and Settings

The first dataset we use is downloaded from Google Earth, which has 200 images with spatial resolutions ranging from 1 m–5 m. The sizes of these images range from 978×586–2535×2297. These images include many different kinds of terrain around the urban area, such as plains, hills, mountains, ports, forests, farmland, and so on. The second dataset we choose is a DigitalGlobe dataset (available at: http://blog.digitalglobe.com/news/open-imagery-and-data-to-support-ecuador-earthquake-response/), which is an open imagery dataset to support Ecuador earthquake response. The main difference of this dataset is that it contains original remote sensing images captured for the purpose of important event applications. After choosing cloudless images containing built-up areas, in our experiment, we use 40 images with a spatial resolution of 0.5 m, whose side length ranges from 1000–2500. For each dataset, out of all images, half of the images are randomly chosen as training samples, and the rest are used for testing. To avoid unnecessary deviation, we repeat the procedure 10-times, which are then averaged. Since we also use the unsupervised methods [13,15,16] for comparison, which do not require training data, we use all images to evaluate their performance.

To quantify the urban area detection performance of different methods, we adopt four widely-accepted evaluation measures, which are OA, kappa coefficient, TPR and FPR [15,49,50,51]. The definitions of TPR and FPR are: TPR=TP/(TP+FN), FPR=FP/(FP+TN), where TP denotes the number of correctly detected pixels in urban area, TN denotes the number of correctly detected pixels in non-urban area, FP denotes the number of wrongly detected pixels in urban area and FN denotes the number of wrongly detected pixels in the non-urban area. Overall accuracy (OA) reflects the ratio of properly classified pixels to the total number, and the kappa coefficient measures the consistency of the result distribution. The formula definition of OA and kappa coefficient can be referred to [49]. In general, larger OA, kappa coefficient, TPR and smaller FPR imply better urban area detection performance.

In order to obtain samples from VHR images, a square patch is used to extract deep features. To process cross-regional patches and assign a proper patch category in the training, we crop a four-times larger square box surrounding the patch and adopt the majority label as the patch label. The DCNNs proposed by Alex et al. are used for feature extraction, with an implementation provided within the Caffe framework [38]. The original pre-trained network model is directly used here, which contains 5 convolutional layers with ReLU activation function, 3 pooling layers with max pooling and approximate 60Mparameters in total. The only minor revisions we have made to use the pre-trained network are that we resize the image patch to accord with the network input and take the first fully-connected layer as the feature extraction output (abandon the rear softmax layers). We simplify the feature extraction process and leave the more time-consuming training for the following classification part. The centroid number of K-means clustering is set to 60 according to the literature [23] to achieve a good balance between proper quantization, overfitting and time. The threshold of urban area decision will be discussed in the following section.

### 3.2. Results on the Google Earth Dataset

We first test our method on the Google Earth dataset. To this end, we first consider the parameter settings. There are mainly three parameters that need to be tuned in our proposed method, i.e., the prior probability of a patch that belongs to an urban area α, image patch size *n* and threshold η. In the experiments, we set α=0.5. For the image patch size *n*, it is difficult to detect the built-up area when n≤10, and it would degrade the detection performance with too large of an image patch size. Table 1 and Table 2 show the performances of the proposed method before and after post-processing for different η when *n* = 15 and 21, respectively. It can be clearly seen that reducing the threshold η helps to improve the TPR. However, the FPR increases at the same time because some non-urban areas are misclassified into urban areas. From Table 1, it can be observed that the TPR and FPR remain the same when η ranges from 0.65–0.80, which indicates that a small image patch is not enough to extract discriminative features to distinguish between urban area and non-urban area. In addition, the OA and kappa coefficient first increase with the threshold η and then begin to decrease after reaching a peak value. Moreover, it can be clearly seen from Table 1 and Table 2 that the post-processing step can actually improve the urban area detection performance.

**Figure 4:**
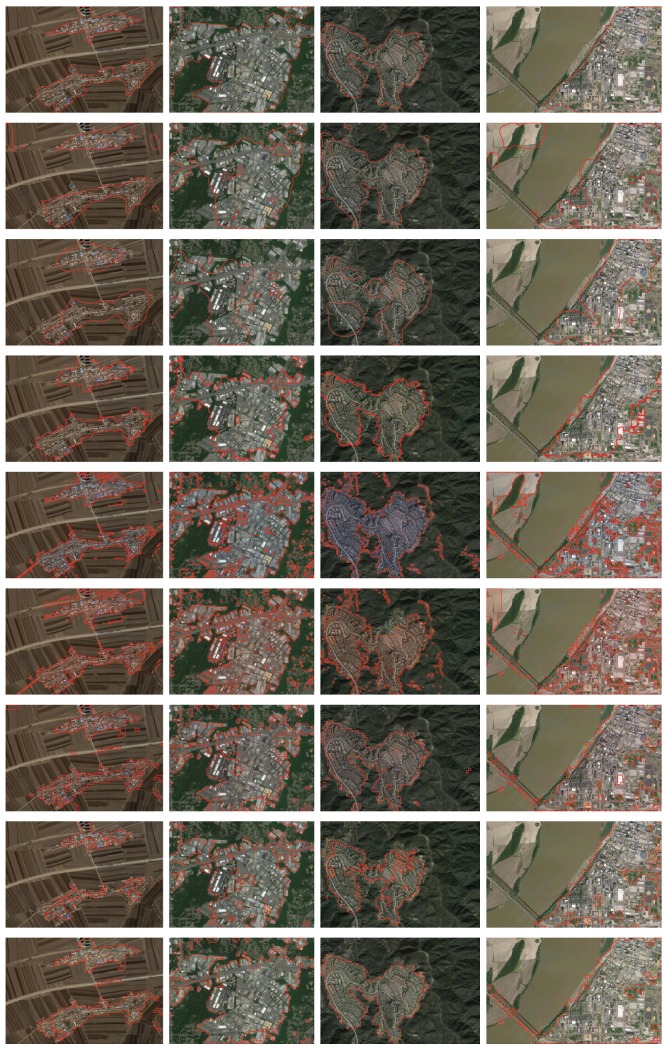
Qualitative illustration of different methods for urban area detection on four typical images of the Google Earth dataset. From left to right: #3 image (43.845 N, 125.084 E, 1.636 m/pixel, 952 × 1406), #7 image (35.271 N, 80.821 W, 2.905 m/pixel, 952 × 1236), #31 image (34.075 N, 118.551 W, 1.943 m/pixel, 952 × 1426) and #42 image (35.134 N, 90.064 W, 2.207 m/pixel, 952 × 1568). From top to bottom: ground truth, results of the method in [13], the method in [15], the method in [16], visual word region detection (VWRD) [23], PCA + SVM, DCNNs + SVM, CNN [36] and our proposed method.

To get an intuitive impression of our method’s performance, we demonstrate the qualitative detection results on four typical images involving different kinds of terrains around the urban area, as shown in Figure 4. The results of the other seven methods are also provided in the figure for comparison. From the results, we see that the method in [13] is able to identify most of the urban areas, while the method in [15] is able to label most of the non-urban areas, but neither of them can accurately distinguish urban and non-urban areas at the same time. The method in [36] seems to show good TP, but FN is higher than the other supervised method. However, it is clear that our method can strike a good balance between identifying the urban and non-urban areas, which achieves results fairly consistent with the ground truth.

**Figure 5:**
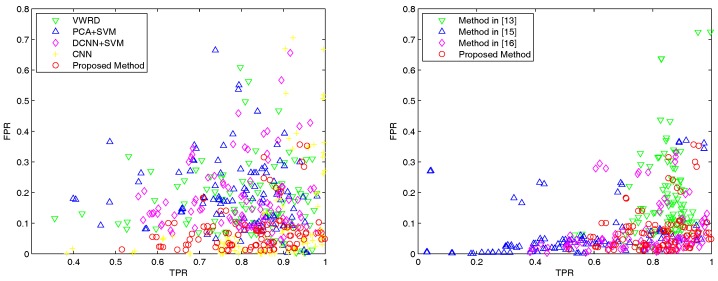
The results of different methods for urban area detection on the Google Earth dataset, where each scatter point indicates a pair of TPR and FPR. Left: Performance comparison of the proposed method and compared supervised methods. Right: Performance comparison of the proposed method and compared unsupervised methods. Our method is indicated by red circles scattered in the bottom right corner.

Next, we report the quantitative results of the proposed method and the other five compared methods on the whole dataset. We select image patch size n=21 and threshold η=0.65, which can obtain the best OA and kappa coefficient, as well as strike a balance between the TPR and FPR. In the left subfigure of Figure 5, it shows the overall performances of the proposed method and four supervised compared methods including VWRD [23] PCA + SVM, DCNNs + SVM and CNN [36]. From the results, it can be observed that our proposed method shows the best consistency, and CNN achieves good TPRs and some high FPRs. These neural network-based methods get obviously lower FPRs than the other methods, which demonstrates that the CNNs can extract richer features from images.

The right subfigure of Figure 5 shows the overall performances of the proposed method and the three compared unsupervised methods in [13,15,16]. It can be seen that the number of best FPRs of the proposed method is comparable with that of the method in [15], while the proposed method clearly shows better TPRs than the method in [16]. The mean TPR of the method in [15] is obviously smaller than the other methods, since it is difficult to correctly detect all the corners, and it performs even worse when noise exists in the image. The extracted region is correlated with the distribution of corners in built-up regions, which may lead to errors on regions’ edges if corners are not detected precisely.

Table 3 shows the average performances of the proposed method and the other seven compared methods for urban detection on the whole dataset. It can be clearly seen that our proposed method obtains the best OA and kappa coefficient and the third best FPR and TPR. The method in [13] has a good TPR and the worst FPR. The method in [15] has a good FPR, but it obtains the worst TPR. The FPR of the SVM classifier is very high probably because it misclassifies some similar features between urban area and non-urban area. The results of VWRD deserve more attention since this method is based on bag of words, which has the same classification approach as we adopt. The different part of VWRD and our proposed method is the feature extraction step: VWRD extracts features by PCA, and we implement with DCNNs. Our proposed method and VWRD have better OA and kappa coefficient than DCNNs + SVM and PCA + SVM, respectively, which demonstrating that the bag of words approach performs better in the area classification step than SVM. Furthermore, since our proposed method and DCNNs + SVM have better OA and kappa coefficients than VWRD and PCA + SVM, respectively, this validates that the DCNNs can extract much richer features from the data automatically compared to PCA, which are more distinct between urban area and non-urban area. The method in [36], which trains a deep end-to-end CNN with several convolutional layers on the training set, also shows good performance. The trained CNN with the softmax classifier helps it to obtain the amazing TPR and fairly the other good indices.

### 3.3. Results on The Digital Globe Dataset

We further test our method on the DigitalGlobe dataset. Compared with the first dataset, this can be seen as an evaluation of a small sample condition. The same parameters are set as those in the above experiments. More specifically, Table 4 and Table 5 show the performances of the proposed method before and after post-processing for different η when *n* = 15 and 21, respectively. It is validated again that when the threshold η increases, the TPR generally decreases, while the FPR generally increases, implying that the non-urban areas are misclassified into urban areas. When n=21 and threshold η=0.7, the proposed method can obtain the best OA and kappa coefficient, and it can also strike a balance between the TPR and FPR. Moreover, the post-processing step can improve the urban area detection performance, which demonstrates the efficiency of incorporating the spatial consistency to improve the detection performance.

**Figure 6:**
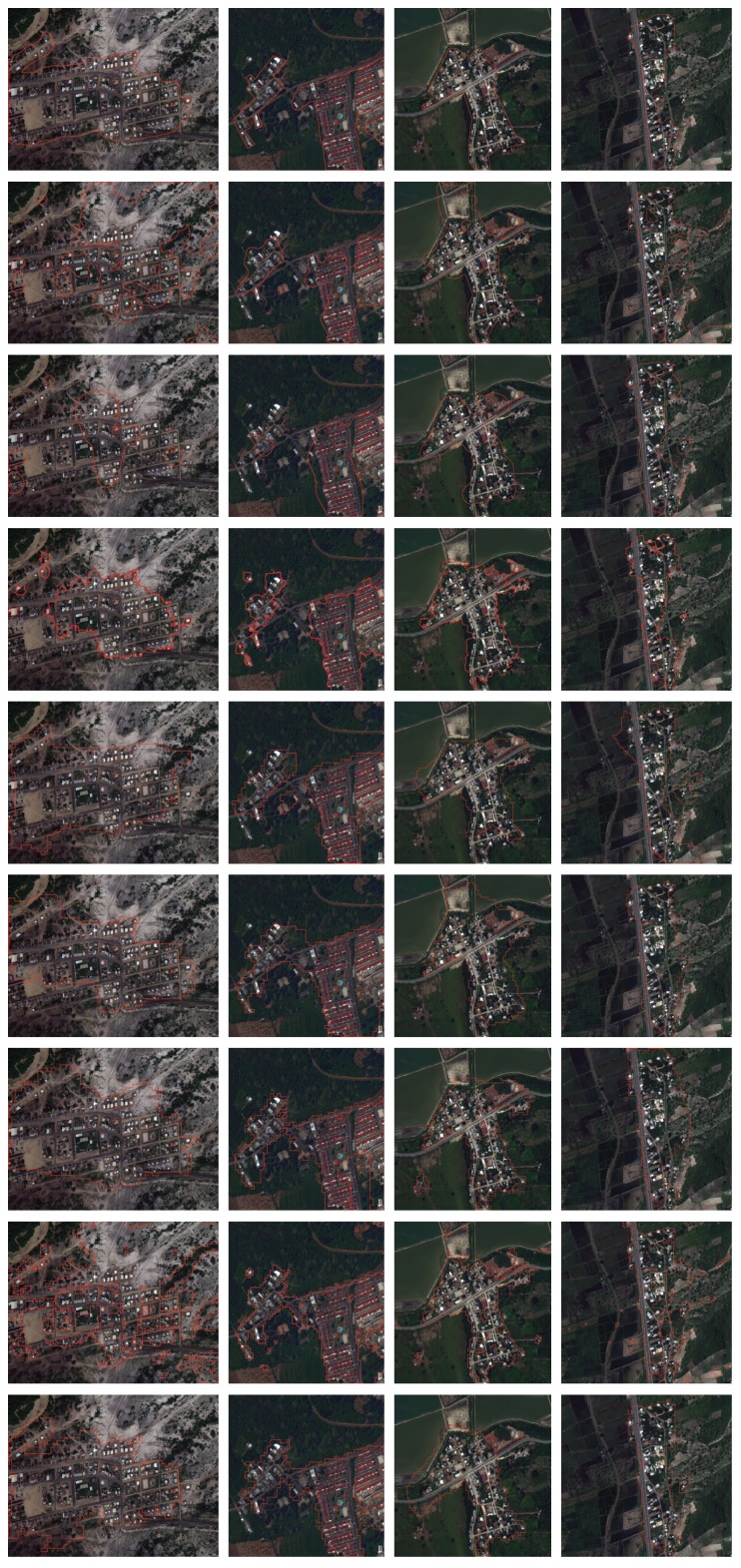
Qualitative illustration of different methods for urban area detection on four typical images of the DigitalGlobe dataset. From left to right: #1 image (2.417 S, 80.606 W, 0.5 m/pixel, 1352 × 1044), #5 image (1.033 N, 80.455 W, 0.5 m/pixel, 1020 × 1064), #10 image (0.633 N, 80.356 W, 0.5 m/pixel, 1336 × 1384) and #38 image (0.917 N, 80.467 W, 0.5 m/pixel, 1608 × 1532). From top to bottom: ground truth, results of the method in [13], the method in [15], the method in [16], VWRD [23], PCA + SVM, DCNNs + SVM, CNN [36] and our proposed method.

To get an intuitive impression of the proposed method’s performance, we also give four representative images for visual comparison. Figure 6 shows the urban detection performances of the compared methods and the proposed method. It can be clearly seen that the urban detection performances of two unsupervised methods [13,15] are worse than the other five supervised methods. The method in [16] shows better overall accuracy, while some urban areas are not detected. Among the supervised methods, VWRD and PCA + SVM intend to enclose more non-urban areas, and methods with DCNNs approximate the best to the ground truth. CNN performs poorly under this evaluation, since many patches are misclassified.

**Figure 7:**
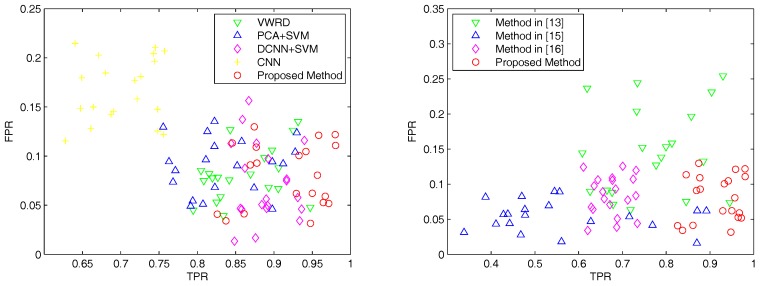
The results of different methods for urban area detection on the DigitalGlobe dataset, where each scatter point indicates a pair of TPR and FPR. Left: Performance comparison of the proposed method and supervised compared methods. Right: Performance comparison of the proposed method and unsupervised compared methods. Our method (red circles, bottom right corner) has the best TPR and FPR overall.

We subsequently give quantitative results of the proposed method and the other seven compared methods using the DigitalGlobe dataset. Figure 7 shows the results of different methods for urban area detection involving all the images, where each scatter point indicates a pair of TPR and FPR. As shown in Figure 7, the performance of our proposed method outperforms all unsupervised methods (right subfigure). In the left subfigure, though the advantage decreases, it is also obvious enough to point out the best approach. Unexpectedly, the method with CNN fails to yield good results this time, and the average performances shown in Table 6 also reveal a similar problem. According to our analysis, under the circumstance that limited samples can be provided, the end-to-end CNNs may be ill-trained, and the non-linear layers may be over-fitted. On the contrary, DCNNs that have been pre-trained on vast labeled natural images perform much better on this dataset, which validates that our selection of pre-trained DCNNs in the pipeline is versatile.

## 4. Conclusions

In conclusion, we propose a new urban area detection method based on the DCNNs and bag of visual words. It involves three major parts including constructing a visual dictionary based on pre-trained DCNNs, learning urban words from labeled images and detecting urban regions in a test image. The overall performance of the proposed method is generally better than other involved state-of-the-art methods, which demonstrates that the combination of DCNNs and bag of visual words detection is superior to other urban area detection pipelines. Our proposed scheme is able to distinguish urban and non-urban areas accurately under small sample conditions, which shows great potential in many remote sensing applications.

## Figures and Tables

**Table 1:** Performances of the proposed method before and after post-processing for different η when *n* = 15 using the Google Earth data.

η	Before P.P.				After P.P.			
	TPR	FPR	OA	Kappa	TPR	FPR	OA	Kappa
0.50	0.7514	0.1711	0.7971	0.5805	0.8712	0.1606	0.8524	0.6997
0.55	0.7360	0.1411	0.8085	0.6005	0.8459	0.1426	0.8527	0.6979
0.60	0.7125	0.1267	0.8073	0.5953	0.8339	0.1163	0.8633	0.7175
0.65	0.6310	0.0713	0.8066	0.5834	0.7098	0.0828	0.8321	0.6438
0.70	0.6310	0.0713	0.8066	0.5834	0.7098	0.0828	0.8321	0.6438
0.75	0.6310	0.0713	0.8066	0.5834	0.7098	0.0828	0.8321	0.6438
0.80	0.6310	0.0713	0.8066	0.5834	0.7098	0.0828	0.8321	0.6438
0.85	0.3253	0.0301	0.7055	0.3266	0.3728	0.0343	0.7225	0.3711
0.90	0.2679	0.0114	0.6930	0.2879	0.3544	0.0237	0.7212	0.3646

**Table 2:** Performances of the proposed method before and after post-processing for different η when *n* = 21 using the Google Earth data.

η	Before P.P.				After P.P.			
	TPR	FPR	OA	Kappa	TPR	FPR	OA	Kappa
0.50	0.7654	0.1532	0.8133	0.6137	0.8576	0.1259	0.8673	0.7276
0.55	0.7143	0.1078	0.8189	0.6187	0.8267	0.1048	0.8670	0.7245
0.60	0.6709	0.0670	0.8250	0.6258	0.8067	0.0768	0.8752	0.7392
0.65	0.6756	0.0434	0.8408	0.6580	0.8092	0.0740	0.8779	0.7447
0.70	0.6193	0.0533	0.8118	0.5930	0.7411	0.0498	0.8640	0.7113
0.75	0.5171	0.0377	0.7788	0.5123	0.6275	0.0442	0.8205	0.6116
0.80	0.4882	0.0303	0.7713	0.4924	0.5887	0.0230	0.8170	0.5996
0.85	0.3263	0.0200	0.7106	0.3390	0.4563	0.0209	0.7637	0.4716
0.90	0.2507	0.0204	0.6792	0.2581	0.3374	0.0213	0.7144	0.3491

**Table 3:** Average performances of different methods using the Google Earth dataset.

	OA	Kappa	TPR	FPR
Method in [13]	0.8141	0.6258	0.8345	0.2015
Method in [15]	0.7465	0.4571	0.4988	0.0640
Method in [16]	0.8535	0.6819	0.7538	0.0599
VWRD [23]	0.8223 ± 0.0014	0.6350 ± 0.0025	0.8115 ± 0.0039	0.1705 ± 0.0047
PCA + SVM	0.8002 ± 0.0039	0.5904 ± 0.0081	0.7891 ± 0.0098	0.1921 ± 0.0062
DCNNs + SVM	0.8155 ± 0.0044	0.6212 ± 0.0079	0.8024 ± 0.0021	0.1755 ± 0.0081
CNN [36]	0.8751 ± 0.0025	0.7345 ± 0.0033	0.8658 ± 0.0033	0.1074 ± 0.0053
Proposed method	0.8765 ± 0.0011	0.7408 ± 0.0012	0.8195 ± 0.0028	0.0778 ± 0.0019

**Table 4:** Performances of the proposed method before and after post-processing for different η when *n* = 15 using the DigitalGlobe data.

η	Before P.P.				After P.P.			
	TPR	FPR	OA	Kappa	TPR	FPR	OA	Kappa
0.50	0.7654	0.1532	0.8133	0.6137	0.8576	0.1259	0.8673	0.7276
0.55	0.7143	0.1078	0.8189	0.6187	0.8267	0.1048	0.8670	0.7245
0.60	0.6709	0.0670	0.8250	0.6258	0.8067	0.0768	0.8752	0.7392
0.65	0.6756	0.0434	0.8408	0.6580	0.8092	0.0740	0.8779	0.7447
0.70	0.6193	0.0533	0.8118	0.5930	0.7411	0.0498	0.8640	0.7113
0.75	0.5171	0.0377	0.7788	0.5123	0.6275	0.0442	0.8205	0.6116
0.80	0.4882	0.0303	0.7713	0.4924	0.5887	0.0230	0.8170	0.5996
0.85	0.3263	0.0200	0.7106	0.3390	0.4563	0.0209	0.7637	0.4716
0.90	0.2507	0.0204	0.6792	0.2581	0.3374	0.0213	0.7144	0.3491

**Table 5:** Performances of the proposed method before and after post-processing for different η when *n* = 21 using the DigitalGlobe data.

η	Before P.P.				After P.P.			
	TPR	FPR	OA	Kappa	TPR	FPR	OA	Kappa
0.50	0.8344	0.1770	0.8263	0.6082	0.9171	0.1427	0.8739	0.7110
0.55	0.7915	0.1170	0.8543	0.6660	0.8921	0.1114	0.8896	0.7444
0.60	0.7265	0.0752	0.8568	0.6721	0.8708	0.0974	0.8931	0.7518
0.65	0.7408	0.0626	0.8702	0.7015	0.8791	0.0902	0.9007	0.7686
0.70	0.6823	0.0683	0.8421	0.6416	0.8079	0.0650	0.8941	0.7539
0.75	0.5996	0.0562	0.8088	0.5753	0.6941	0.0666	0.8483	0.6547
0.80	0.5619	0.0463	0.7929	0.5465	0.6551	0.0438	0.8435	0.6466
0.85	0.4069	0.0405	0.6878	0.3698	0.5255	0.0456	0.7713	0.5066
0.90	0.3302	0.0341	0.6192	0.2783	0.4152	0.0451	0.6929	0.3758

**Table 6:** Average performances of different methods using the DigitalGlobe dataset.

	OA	Kappa	TPR	FPR
Method in [13]	0.8381	0.6048	0.8094	0.1522
Method in [15]	0.8264	0.4828	0.4900	0.0602
Method in [16]	0.8459	0.5944	0.6545	0.0760
VWRD [23]	0.8872 ± 0.0054	0.7600 ± 0.0023	0.8560± 0.0042	0.0876 ± 0.0037
PCA + SVM	0.8755 ± 0.0042	0.7535 ± 0.0056	0.8528 ± 0.0038	0.0901 ± 0.0053
DCNNs + SVM	0.8889 ± 0.0035	0.7766 ± 0.0061	0.8970 ± 0.0025	0.0845 ± 0.0036
CNN-2 [36]	0.8046 ± 0.0028	0.5244 ± 0.0037	0.7046 ± 0.0025	0.1648 ± 0.0033
Proposed method	0.9212 ± 0.0016	0.7998 ± 0.0015	0.9100 ± 0.0017	0.0751 ± 0.0026
